# Analysis of factors affecting students going to school toilets in a rural primary school in China

**DOI:** 10.1186/s12889-020-10099-4

**Published:** 2021-01-06

**Authors:** Tang Shao, Jingjing Zhao, Haijuan Hu, Qi Zhang

**Affiliations:** 1 National Center for Rural Water Supply Technical Guidance, Chinese Center for Disease Control and Prevention, 13 Government Street, Changping District, Beijing, China; 2 Chongqing Center for Disease Control and Prevention, 8 Changjiang Second Road, Yuzhong District, Chongqing, China

**Keywords:** Behavior, China, Cleanliness, Experience, Students, Toilet use

## Abstract

**Background:**

Several factors may affect students going to school toilets, but a few studies have analyzed the reasons for students using toilets. This study aimed to use a structural equation model to understand the factors that impacted children’s toilet behavior.

**Methods:**

This study was performed in 12 rural nonboarding primary schools (6 schools in the northern and 6 schools in the southern regions of China). All students of the third and sixth grades (761 students) were examined. A questionnaire on students’ toilet behavior was used. The questionnaire included 33 perceptual items based on 5 factors: toilet facilities, cleanliness, hygiene practices, peer relationship, and experience. The questionnaire also covered the frequency of voiding and defecating by themselves. The exploratory factor analysis, confirmatory factor analysis, and pathway analysis were used to analyze the causes of students’ toilet behavior.

**Results:**

A statistically significant correlation coefficient of 0.300 indicated that cleanliness impacted the toilet frequency of students. The visual experience of the overall cleanliness of the toilet had the most significant impact on students’ toilet behavior (path coefficient, 0.81). Washing facilities and convenient handwashing had the least impact on toilet use (path coefficient, 0.52).

**Conclusion:**

Cleanliness was the primary consideration for students’ toilet use on campus. The visual experience of the overall cleanliness of toilets had the most significant impact when students used toilets. No pre-survey was conducted to test the reliability and validity of the questionnaire. Using self-reported data might be associated with potential recall errors.

**Supplementary Information:**

The online version contains supplementary material available at 10.1186/s12889-020-10099-4.

## Background

A survey of school children in developed countries found inadequate toilet facilities in schools and the reluctance of children to use them. Data for 2005 showed that 25% of students had constipation and 26% never used school toilets to defecate [1]. These data might seem a little outdated due to the lack of the latest research, but they did reflect problems with students’ toilet behavior. These studies gave every reason to believe that students’ hygiene might be worse in less developed areas. Healthy hygiene habits can be best inculcated since the early years of childhood. However, past studies suggested that traditional didactic health education was unlikely to be a valid route to behavioral change [2]. The reason for this problem might be that hygiene behaviors were rarely examined for health-related reasons for ordinary people, especially children.

In contrast, in the non-educational environment, the school’s comprehensive WASH (Water, Sanitation, Hygiene) interventions might significantly improve students’ hygiene [3]. According to the World Health Organization, 11% more girls attended schools when sanitation was available [4]. The evidence on the impact of improved school washing on health and education was limited, but it was indeed convincing. Studies in China and Kenya showed that school health promotion campaigns could reduce absenteeism due to sickness by 20–58%, and could also reduce absenteeism among girls [3, 5].

Toilet-related behaviors were one of the most critical hygiene behaviors in protecting health. Healthy behaviors of using school toilets were critical because voiding postponement incontinence was associated with a low micturition frequency, urgency, and behavioral problems [6]. The data showed that children voided 2–10 times per 24 h (median 5), and most (95%) avoided voiding at a frequency of 3–8 times [7]. Children aged 7–12 years in a Japanese primary school urinated about five or six times daily [8]. However, a study examined 385 Swedish school children aged 6–16 years and revealed that 25% (overall 16%) of children reported never using the school toilet to urinate, and 80% (overall 63%) never used it to defecate [9]. When children suppressed or ignored “full-bladder” signals, the risk of developing emptying disturbances and urinary tract infections increased [9]. A timed voiding schedule was essential in treating dysfunctional voiding [10]. Thus, analyzing the causes affecting students going to school toilets was necessary.

Children aged 7–15 years often based their decision to relieve themselves on behavioral and social factors [7]. Nevertheless, a limited number of studies focused mainly on behaviors of students using school toilets. Some studies suggested several possible causes affecting students going to school toilets. For example, one cluster randomized trial, including latrine provisions, evaluated the influence of school WASH on health and absenteeism [11]. Results from in-depth interviews showed that students would weigh multiple factors to decide whether to use the school toilet. The factors included physical environmental factors (conditions, safety, privacy, accessibility, and availability), social factors (norms, expectations, and responsibility), and individual factors (experience, routine, risk perception, and personal needs) [12]. It implied that factors such as washing and cleaning toilets, making them smell good, removing dirty contaminating matter from facilities, and protecting children’s privacy might be attractive factors for students to use school toilets.

However, some studies analyzed these causes and the extent to which they impacted children’s toilet behavior. Studies investigating the possible factors and the priority factors were also limited. Thus, this study aimed to use a structural equation model to understand the factors that impacted children’s toilet behavior. The conclusion of this study also provided a reference for school health intervention. This study involved a qualitative survey in two counties in rural China, one in the north and another in the south.

## Methods

### Sample size and field sites

This was a cross-sectional study. Referring to the researches on qualitative variables in cross-sectional surveys, the following formula ([13] was used to estimate the population parameters:

$$ \mathrm{Sample}\ \mathrm{size}=\frac{{Z_{1-\alpha /2}}^2p\left(1-p\right)}{d^2} $$

where *Z*_1 − *α*/2_ is a standard normal variable. As in the majority of studies, *P* values less than 0.05 were considered significant. Hence, 1.96 was used in the formula (*P* = expected proportion in population-based previous studies). This study referred to an extensive survey on the use of toilets by British students because studies on the use of school toilets in China were limited [14]. The results showed that 40% of students never used the school toilet to defecate. Therefore, the value of *P* in the aforementioned formula was 0.4 (*d* = absolute error of precision). The sample size was calculated with an absolute error of 5% and a type 1 error of 5%. Therefore, using the formula, the sample size was 369. Hence, at least 369 participants were required for this cross-sectional study.

When choosing schools, several causes needed to be considered [1]. Nonboarding schools including only grades 1–6 were included. Boarding schools, schools with incomplete grades 1–6, and schools including high school grades were excluded [2]. Schools with complete WASH facilities were included referring to the national rural school construction standards, *Code for design of school* [3, 15]. Schools with a high degree of cooperation among school administrators were included. Each grade in rural schools had about 30 registered students, and on-site surveys had missing samples. Finally, 12 schools were included in the survey, 6 schools located in north China and 6 in south China.

### Imposing a theoretical framework

Many students did not use the school toilet to urinate and defecate [9]. The problem might be the result of a combination of multiple factors. Nevertheless, previous studies mostly focused on a single reason. The widespread impact of the cause on students’ toilet behavior in school and the independence of interaction between the causes needed further exploration.

Previous studies found that some reasons might impact students’ toilet behavior in school. These reasons included toilet facilities ([10, 16–18]), cleanliness [19–22], hygiene practice [23, 24], peer relationship [12, 25, 26], and experience [27–29].

**Fig. 1:**
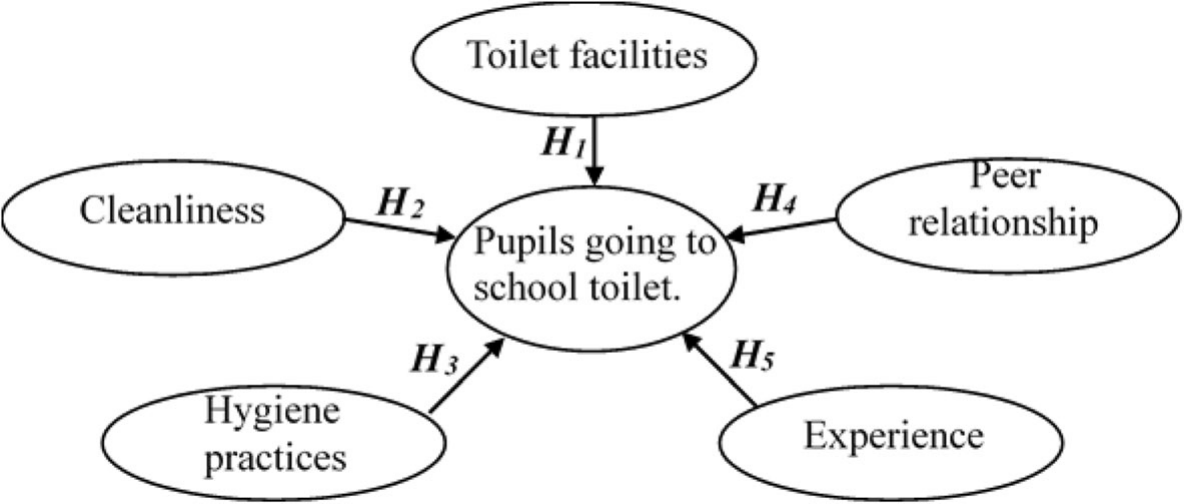
A theoretical framework of students going to a school toilet. ***H***_***1***_–***H***_***5***_ represent the hypothesis that each common factor had a direct and significant impact on students going to a school toilet

Based on previous findings, this study proposed a theoretical framework (Fig. 1) to describe the factors that impacted children’s toilet behavior in school.

The theoretical framework was used to guide some designs in this study. For example, it guided the designing of the questionnaire (e.g., designing practical issues around the influencing factors), guided data analysis [e.g., analysis of survey data by structural equation modeling (SEM)], and helped in the interpretation of results (e.g., to verify research hypotheses based on the theoretical framework).

### Questionnaire development

Although the quality of hygiene facilities affected students’ toilet behavior [3], the subjective experience of using the toilet would also influence students’ toilet behavior [22]. This study focused more on the impact of sanitation of school toilets on students’ toilet behavior. Based on the theoretical framework, previous findings [10, 14, 30] were also referred. A questionnaire, including five factors, was developed: toilet facilities (number of toilets, distance from classroom to toilets, and so forth), cleanliness (floor, defecation pit, and so forth), hygiene practices (washing hand behavior, awareness of using a toilet, and so forth), peer relationship (number of friends, relationship with friends, and so forth), and experience (meeting people who scare you). The questionnaire included 33 perceptual items in total (Additional files). The answer to each item was designed to be five degrees from most optimal to least optimal, referring to the designs of Likert scale questions. Moreover, the self-reported frequency of voiding and defecating was also included. The study protocols and questionnaire were reviewed by the Ethics Committee of National Center for Rural Water Supply Technical Guidance, Chinese Center for Disease Control and Prevention.

### Data collection

Each school had to have at least one class of students to ensure a sufficient sample size. Although students fully understood the questionnaire content, they refused to participate in the survey for other reasons. The survey selected third-grade and sixth-grade students from each school. The questionnaire developed in this study was used to investigate the students’ toilet behavior. After obtaining consent from participants, the one-on-one interview method was used to help students understand the questions correctly and avoid inauthentic answers. This study employed trained professionals in environmental health and health education fields to help conduct the survey. At the same time, the questionnaire was not handed over to students and teachers in advance. This survey was conducted in June 2019. The collected data were entered using Excel (version 2010) by two persons parallelly to avoid mistakes. Subsequently, the data were logically reviewed before carrying out the analysis.

### Analysis

#### Demographic characteristics of students and survey data presentation

This study used the number and proportion to describe the demographic characteristics of the sample population and the frequency of urination and defecation. Besides, the study also used the number and proportion to describe the result of each item of the questionnaire. Students of different ages and sexes might have differences in their choices of using toilets in the school. However, this study focused on analyzing the impact of students’ experience on their toilet behavior. Therefore, a correlation analysis was performed between the toilet experience of students and the frequency of toilet use.

#### Exploratory factor analysis

The data might be missing on a few items in the questionnaire due to the use of the one-to-one survey method. On the contrary, the analysis method using SEM needed the data to be complete. Therefore, the answer to each item in the questionnaire was assigned 1–5 points from the most optimal to least optimal, and the missing value was filled using the mean substitution method.

A principal component analysis (PCA) was carried out using SAS (version 9.4) on the survey data. The maximal rotation of variance was used to preserve the factors whose root eigenvalue was more significant than 1. An exploratory factor analysis (EFA) was carried out using all the items. The Kaiser–Meyer–Olkin (KMO) value and the Bartlett spherical test value of the questionnaire data were calculated; the preliminary analysis showed the data were suitable for factor analysis [31]. Items with a factor load of less than 0.4 [31, 32] was rejected, and the standard for the cumulative variance contribution rate was higher than 0.5 [31]. Based on the result of the factor load, the items of the questionnaire were screened.

#### Confirmatory factor analysis

This study used confirmatory factor analysis (CFA) to test the latent variables proposed using the theoretical framework. All SEM analyses were performed with Amos, version 7.0, using maximum likelihood estimation with standard errors and parameter coefficients of the SEM. A *P* value < 0.05 (two-sided) was the level of statistical significance [33]. This study used the *χ*^2^ test and some indicators of model fit to assess the model fit. The indicators of model fit included normed fit index, comparative fit index (CFI), and root mean square error of approximation (RMSEA) [34].

Additionally, the Akaike Information Criterion (AIC) was also used to assess the model [35]. However, no overall test of model fit was available for such a model, in which case it was recommended to prefer the model with the smallest AIC value [35].

#### Path analysis with variables

**Fig. 2:**
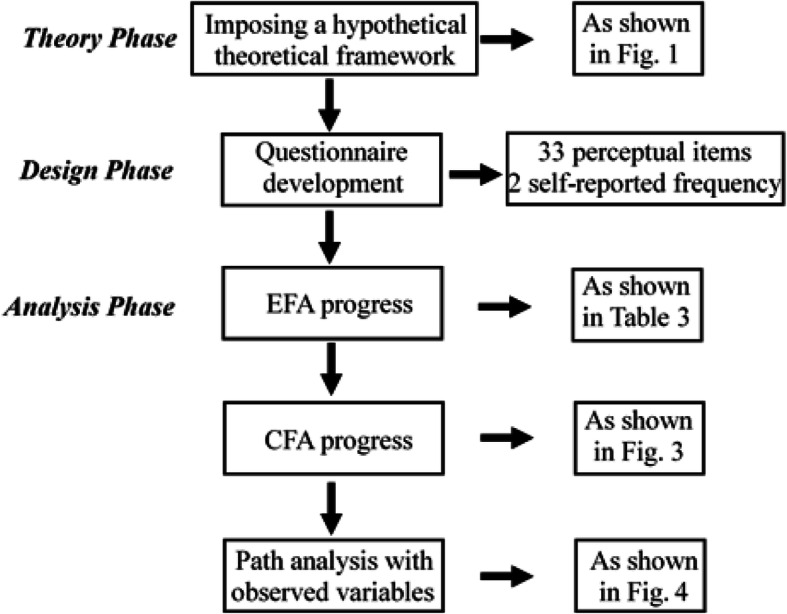
Data collection and analysis progress in the study

The validity of the questionnaire was tested by EFA and CFA. Based on the result, path analysis with variables [33] was used to analyze the effect of hypothetical factors on school toilet behaviors of students. Indexes such as *χ*^2^ value, CFI, AIC, and others [34, 35] were also used to guide the model correction. The progress of the analysis is shown in Fig. 2.

## Results

### Sample description

Table 1 presents the distribution of demographic characteristics of students and the frequency of students voiding and defecating. A total of 761 students were given the questionnaire, which included 50% each of boys and girls mostly in the age range of 9–14 years. The recovery rate was 100%. The data indicated that more than 90% of students used the school toilet to urinate at a frequency of 3–6 per day. Nearly half of the students occasionally defecated in the school toilet. More than 16% of students never used the school toilet for defecating.

**Table 1:** Demographic characteristics of students and frequency of voiding and defecating in school toilets^a^ (*n* = 761)

Characteristics	No. of participants interviewed	Proportion, %
Sex		
Boys	382	50.20
Girls	379	49.80
Age, year		
≤9	104	13.70
10	171	22.53
11	106	13.97
12	160	21.08
13	107	14.10
≥14	111	14.62
Grade		
Third grade	380	49.93
Sixth grade	381	50.07
Frequency of voiding		
3–4 times per day	401	52.83
5–6 times per day	295	38.87
1–2 times per day	39	5.14
≤1 time per day	17	2.24
Never	7	0.92
Frequency of defecation		
Sometimes	366	48.16
Always	267	35.13
Never	127	16.71

^a^Numbers might not sum to a total because of missing data

Table 2 presents the number and proportion of each choice for each item in the toileting-related questionnaire. Choice a to choice e represented five degrees from most optimal to least optimal.

**Table 2:** Number and proportion of each option from variables in the students’ behavior of using the questionnaire^a^ (*n* = 761)

Variable	Proportion of each option from a variable (*n*)				
	Option a	Option b	Option c	Option d	Option e
**Toilet facilities**					
Was the toilet far away	86.55 (650)	12.52 (94)	0.67 (5)	0.13 (1)	0.13 (1)
Was the time enough to use the toilet at break	97.21 (731)	2.26 (17)	0.53 (4)	0	0
Was the toilet usually crowded	71.35 (538)	24.27 (183)	3.58 (27)	0.80 (6)	0
Did you need to wait while using the toilet	75.50 (570)	21.85 (165)	1.19 (9)	1.19 (9)	0.26 (2)
Were you late for class due to using the toilet at break	73.33 (550)	26.40 (198)	0.13 (1)	0	0.13 (1)
Were you criticized by a teacher due to using the toilet	91.97 (653)	7.61 (54)	0	0	0.42 (3)
**Toilet hygiene**					
Was the toilet usually clean	53.39 (402)	21.12 (159)	18.73 (141)	6.64 (50)	0.13 (1)
Was there usually any stool or urine on the toilet floor	66.05 (498)	24.67 (186)	3.45 (26)	5.31 (40)	0.53 (4)
Was there usually any garbage (such as toilet paper) on the toilet floor	64.19 (484)	27.85 (210)	2.79 (21)	4.51 (34)	0.66 (5)
Was there usually any dirty water stain on the toilet floor	56.90 (429)	32.49 (245)	3.32 (25)	6.63 (50)	0.66 (5)
Was there usually any stool or urine in the defecation pit	32.85 (247)	36.97 (278)	9.71 (73)	18.62 (140)	1.86 (14)
Was there usually any garbage (such as toilet paper) in the defecation pit	44.15 (332)	28.72 (216)	9.97 (75)	16.36 (123)	0.8 (6)
Was the toilet well ventilated and smell free	31.08 (234)	20.19 (152)	36.92 (278)	11.42 (86)	0.4 (3)
Was the toilet usually dark	65.47 (493)	21.38 (161)	11.16 (84)	1.59 (12)	0.4 (3)
Did you have the experience of slipping or falling in the toilet	87.27 (658)	11.67 (88)	0.66 (5)	0.27 (2)	0.13 (1)
Had you ever accidentally stepped into the defecation pit in the toilet	98.80 (743)	1.06 (8)	0	0.13 (1)	0
Were there usually flies and maggots in the toilet	28.82 (217)	44.49 (33)	15.54 (117)	10.49 (79)	0
Had you ever been bullied by other students in the toilet	91.60 (687)	2.80 (21)	0.2 7 (2)	5.33 (40)	0
**Hygiene practice**					
Would you urinate or defecate on the toilet floor in case of an urgency	98.41 (742)	0.80 (6)	0.13 (1)	0.66 (5)	0
Would you endure waiting for the break if you wanted to use the toilet during class	34.00 (256)	34.26 (258)	12.75 (96)	18.59 (140)	0.4 (3)
Would you litter your used toilet paper	98.01 (738)	0.80 (6)	0	1.20 (9)	0
Would you pay attention to the urine or feces at the designated location in the toilet	84.67 (591)	4.87 (34)	1.43 (10)	8.88 (62)	0.14 (1)
Did the teacher teach you how to wash your hands after using the toilet	18.30 (138)	42.71 (322)	30.37 (229)	6.23 (47)	2.39 (18)
Do you wash hands every time after using the toilet	60.21 (454)	13.53 (102)	18.57 (140)	7.69 (58)	0
**Peer relationship**					
How many close friends did you usually have	74.73 (562)	9.84 (74)	7.71 (58)	7.18 (54)	0.53 (4)
Did you often go to the toilet alone	21.70 (163)	43.28 (325)	25.83 (194)	9.19 (69)	0
Did you usually go to the toilet with close friends	147.3 (108)	44.43 (335)	33.95 (256)	7.29 (55)	0
Would you accompany your close friends to the toilet whenever you did not want to go	26.43 (199)	28.42 (214)	31.87 (240)	13.15 (99)	0.13 (1)
Would you wait for your close friends to return to the classroom whenever you met him or her in the toilet	36.29 (274)	27.81 (210)	28.48 (215)	7.42 (56)	0
**Experience**					
How did you usually deal with the situation of meeting a classmate having a bad relationship with you when going to the toilet	85.66 (645)	6.91 (52)	1.46 (11)	1.86 (14)	4.12 (31)
How did you usually deal with the situation of meeting a classmate who liked to bully other students when going to the toilet	81.56 (615)	7.29 (55)	3.45 (26)	2.25 (17)	5.44 (41)
How did you usually deal with the situation of meeting a classmate who liked to make fun on your going to the toilet	84.69 (636)	7.99 (60)	2.4 (18)	1.46 (11)	3.46 (26)
How did you usually deal with the situation of meeting a teacher when going to the toilet	93.77 (708)	2.91 (22)	1.72 (13)	0.66 (5)	0.93 (7)

^a^Numbers might not sum to a total because of missing data

### CFA progress

#### Questionnaire test and EFA progress

Before the EFA, this study found that a few items had missing data, and the percentage was less than 1%. The PCA method was used to analyze the 33 items. The KMO value of the survey data was 0.848, and the Bartlett sphere test rejected the *H*_0_ hypothesis (*P* < 0.0001). Therefore, the survey data were suitable for factor analysis [36]. Sixteen items were excluded because the factor loading was less than 0.4. Eighteen items were finally retained. Further, the KMO value was 0.856, and the Bartlett sphere test rejected the *H*_0_ hypothesis (*P* < 0.0001).

Because of low factor loading (not reaching 0.4), no valid model could be fitted when the hygiene practice was included in the model. This was probably because of the potential collinearity between hygiene practice and other factors. Thus, the hygiene practice was dropped from the final model (Table 3).

**Table 3:** Results of factor analysis of students going to school toilets^s^

Variable	Cleanliness	Peer relationship	Experience	Toilet facilities
Was the toilet usually crowded (***X***_***1***_)	0.351	− 0.051	0.018	**0.631**
Did you need to wait while using the toilet (***X***_***2***_)	0.182	−0.119	0.035	**0.579**
Was the toilet usually clean (***X***_***3***_)	**0.748**	0.001	0.047	0.204
Was there usually any stool or urine on the toilet floor (***X***_***4***_)	**0.750**	−0.011	0.072	0.152
Was there usually any garbage (such as toilet paper) on the toilet floor (***X***_***5***_)	**0.738**	−0.001	0.023	0.098
Was there usually any dirty water stain on the toilet floor (***X***_***6***_)	**0.636**	0.054	−0.010	0.135
Was there usually any stool or urine in the defecation pit (***X***_***7***_)	**0.757**	−0.089	0.007	0.013
Was there usually any garbage (such as toilet paper) in the defecation pit (***X***_***8***_)	**0.757**	−0.095	−0.030	0.058
Was the toilet usually dark (***X***_***9***_)	**0.497**	−0.110	0.241	0.051
Were there usually flies and maggots in the toilet (***X***_***10***_)	**0.687**	−0.063	0.096	0.139
Did you wash hands every time after using the toilet (***X***_***11***_)	**0.460**	−0.120	0.016	0.233
Did you often go to the toilet alone (***X***_***12***_)	–-0.017	**0.677**	−0.071	−0.174
Did you usually go to the toilet with close friends (***X***_***13***_)	0.002	**0.786**	−0.083	−0.102
Would you accompany your close friends to the toilet whenever you did not want to go (***X***_***14***_)	−0.092	**0.662**	−0.061	−0.022
Would you wait for your close friends to return to the classroom whenever you met him or her in the toilet (***X***_***15***_)	−0.071	**0.558**	−0.051	0.044
How did you usually deal with the situation of meeting a classmate having a bad relationship with you when going to the toilet (***X***_***16***_)	0.065	−0.037	**0.634**	−0.023
How did you usually deal with the situation of meeting a classmate who likes to bully other students when going to the toilet (***X***_***17***_)	0.030	−0.091	**0.635**	0.007
How did you usually deal with the situation of meeting a teacher when going to the toilet (***X***_***18***_)	0.040	−0.074	**0.574**	0.064
Root eigenvalue	5.252	2.469	1.759	1.237
Variance contribution rate (%)	29.178	13.714	9.773	6.872

^a^Bold data indicated that the factor loading was higher than 0.4

Finally, four factors from the theoretical framework were retained, and their cumulative variance contribution rate was 59.54%, which exceeded the standard of 50% [31]. Reliability testing of 18 items found that Cronbach’s alpha value was 0.763, indicating that the questionnaire had the right internal consistency [37]. Cronbach’s alpha values of four items in the model were 0.755, 0.883, 0.761, and 0.700, which explained that each item had good credibility [38]. The reliability was also tested using the average of variance extracted. The values were 0.623, 0.461, 0.472, and 0.437, close to or reaching the reference value of 0.5, implying that each variable could be interpreted using the matching latent variable [39]. The normalized factor loading value was 0.46–0.79; 16 items exceeded 0.5 (Table 3). *H*_0_ indicated that the questionnaire had a better structure [31, 32].

#### CFA progress with research data

This study used EFA to screen out the relevant variables. The SEM method in the CFA helped verify the assumptions. These assumptions included whether these variables were independent of one another, whether these variables could correctly reflect the content of hypothetical factors, and whether any common influence existed between the factors. At the same time, the SEM method also helped verify the validity of the questionnaire. In the SEM method, the chi-square value helped understand whether the model adapting to the data was accepted.

**Fig. 3:**
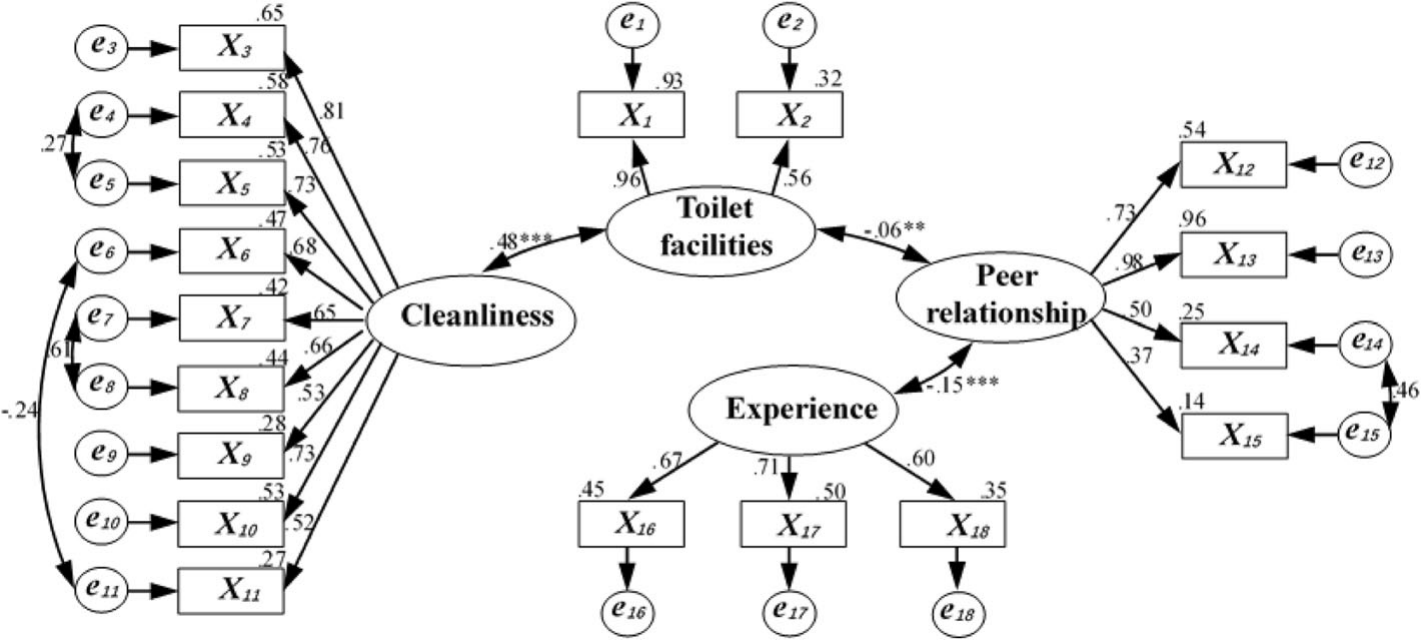
Final SEM and parameter values for the CFA progress. One-way arrows indicate a significant association, and two-way arrows indicate a significant correlation. Numbers over arrows indicated a standardized regression coefficient, and numbers over observed variables (rectangles) indicated explained variance (*R*^2^). *P* < 0.001 and *P* value close to 0.05 were indicated by ^***^ and ^**^, respectively. The *e* indicates the residual in SEM

The results of the EFA progress showed a comparatively slightly better model fit without hygiene practice. Fitting data to the final model (Fig. 3) using weighted least squares estimation yielded a significant *χ*^2^ test of model fit [*χ*^2^ =291.614 (128 df), *P* < 0.000]. It was a signal for model rejection, which was expected because of the enormous sample size [33, 40].

In the SEM method, the model adaptation index helped judge the degree of fit of the model and choose the model with the best fit. The model adaptation index included RMSEA, the adjusted goodness-of-fit statistic (AGFI), the goodness-of-fit index (GFI), the Tucker–Lewis coefficient (TLI), and the critical N (CN). The smaller the RMSEA value, the larger the AGFI, GFI, and TLI values, the better the model fitted. The CN value varied depending on the size of the sample [33, 40].

In the final fitted model, the model adaptation index was RMSEA of 0.041 (90% confidence interval: 0.035–0.047), which was lower than the reference value of 0.08 [33]. The AGFI was 0.945, the GFI was 0.959, and the TLI was 0.963, which were all higher than the reference value of 0.900. CN was 435, which was higher than 200 [33]. These model fit indicators showed a good model fit. According to the SEM model, all factor loadings were more significant than 0.5, except one (peer relationship pointed to *X*_***15***_), which showed that the questionnaire had the right validity. In the SEM model, cleanliness and toilet facilities had a strong correlation (*R*^2^ value was 0.48).

#### Path analysis with research data

Some hypotheses were verified using the CFA and SEM. These hypotheses included the independence between the variables, whether the variables reflected the hypothetical factors, the interaction between the hypothetical factors, and the validity of the questionnaire. However, the impact of hypothetical factors on students’ use of school toilets needed further data analysis. Thus, the SEM method in the path analysis was used to analyze the effect of factors on toilet frequency.

The sum of the scores obtained from the variables, which reflected the same common convergence according to the SEM, represented the factors. The frequency of voiding and defecating was used as a dependent variable. The voiding frequency was divided into five levels: never, ≤1 per day, 1–2 per day, 3–4 per day, and 5–6 per day. The defecation frequency was divided into three levels: always, sometimes, and never. The frequencies, according to the answer, were assigned 1–5 points and 1–3 points, respectively. Finally, the toilet frequency was represented by the scores from voiding and defection frequency.

**Fig. 4:**
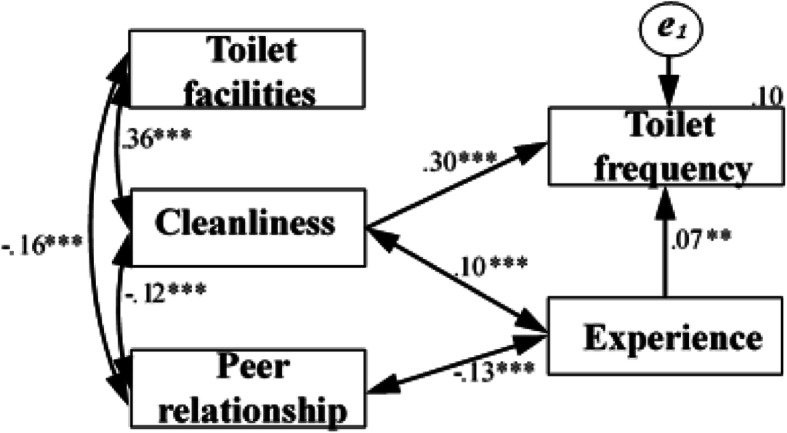
Final SEM and parameter values for path analysis. One-way arrows indicated a significant association and two-way arrows indicated a significant correlation. Numbers over arrows indicated a standardized regression coefficient, and numbers over observed variables (rectangles) indicated explained variance (*R*^2^). A *P* value < 0.001 and a *P* value close to 0.05 were indicated by ^***^ and ^**^, respectively. The *e* indicates the residual in SEM

The final model had a comparatively slightly better model fit (Fig. 4). The model adaptation index was *χ*^2^ value of 5.380 (3 df), *P* = 0.146, RMSEA of 0.032 (90% confidence interval: 0.000–0.076), AGFI of 0.986, GFI of 0.997, TLI of 0.967, and CN of 1590, which showed that the model was within acceptable limits.

The result of the path analysis showed that the frequency of students using school toilets was affected by the cleanliness of the toilets, which supported the *H*_*2*_ hypothesis in the theoretical framework. A certain degree of correlation existed between cleanliness and toilet facilities, which was consistent with the findings of the PCA.

## Discussion

The purpose of this study was to test some determinations imposed by scholars ([12, 16, 21, 26], and so forth) and having an impact on students using school toilets. This study was one of several initial quantitative studies to characterize determinations on toilets based on subjective feelings. Moreover, it provided valuable insights into how to improve students’ toilet use.

The data supported the casual relationship *H*_***2***_ (Fig. 4). Cleanliness was the primary consideration for students’ toilet use on campus, which was consistent with previous findings on the positive effects of cleanliness and students using school toilets [22]. A meaningful and statistically significant association was found between toilet cleanliness and toilet use for both boys and girls [19, 20]. Additionally, providing a clean toilet could significantly reduce the possibility of children being exposed to pathogens [21]. However, toilets lacked measures to deal with feces and urine and handwashing in rural areas in Guangdong and Chongqing, China [41, 42].

Figure 3 shows that the visual experience of the overall cleanliness of the toilet had the most significant impact on students’ toilet use (the path coefficient of 0.81). The washing facilities and convenient handwashing had the least impact on toilet use (the path coefficient of 0.52). Poor toilet conditions, including the presence of feces, urine, blood, vomit, flies, maggots, and smell, led to poor visual and olfactory experience that prevented students from using school toilets [12]. Moreover, children who attended primary schools with better-maintained toilets were less likely to be absent in a cross-sectional study in Kenya [43].

In the final adjusted model, the adequacy of toilet facilities was not an impact factor for students’ toilet use. The crowding did not prevent students from using school toilets when they had physiological needs. Even if the toilet ratios did not reach a relatively sufficient standard, the primary reference standard was the *Code for design of school* [15]. Moreover, this finding was different from previous findings; for example, students were likely not to use toilets when queues were present, particularly during planned breaks [10]. Students had enough break time if they needed to use a toilet, and toilets were usually located close to their study building. Despite queues sometimes, students were not late for classes; hence, crowding had no visible impact on their toilet behaviors. However, the adequacy of toilet facilities did not have a direct and significant impact on students going to school toilets in the study. Enough toilets were required because they were vital in the establishment of healthy voiding habits, prevention of elimination syndromes, and correction of established dysfunctional voiding [10].

The experience of teasing and bullying might be a deterrent to toilet use [12, 25, 26], but it was not reported as a problem in the study. Figure 4 shows the path coefficient of experience pointing to toilet frequency was 0.07. Even if the value was statistically significant, the value was too low. Thus, whether unfriendly experience influenced students using toilets because of students not reporting bullying needed further investigation. This finding was consistent with previous findings [44]. Many students did not disclose bullying they experienced or witnessed because of a sense of helplessness, concerns over inappropriate adult action, self-reliance, shame, and others. Peer relationships were not affected by toilet use in the study, but a negative correlation between peer relationships and experience was consistent with previous findings. For example, the girls said they were “scared” to go to the toilet alone because it was situated away from the primary school buildings, and they faced some problems including lack of privacy, bullying, facing male teachers, and so forth. Thus, they preferred to go to the toilets in pairs [27]. Having good peer relationships could help children avoid these risks when using school toilets.

This study had several limitations. A pre-survey was needed to test the reliability and validity of the questionnaire. Although the sample was big, using an untested questionnaire directly might have missed some details. Because of the limited funds and the shortage of staff to carry out on-site work, this study selected 2 regions and 30 schools with a high degree of coordination to promote the on-site work. Therefore, this study inevitably had a selection bias. Another limitation was the use of self-reported data and the potential for recall errors; students might provide socially desirable answers. Finally, using a structured questionnaire and SEM might be a new trial for assessing hygiene behaviors and others. Testing the influence of multiple possible factors rather than the use of single variables could be more intuitive and convincing. Nevertheless, evidence to prove these findings was not sufficient, and hence more longitudinal designs were needed to support them.

## Conclusions

Cleanliness was the primary consideration for students’ toilet use on campus; the visual experience of the overall cleanliness of the toilet had the most significant impact when students used the toilet. This study was one of several initial quantitative studies to characterize determinations on toilets based on students’ subjective feelings, thus providing some reference for future research.

## Supplementary Information

**Additional file 1.** Questionnaire.

## Data Availability

The datasets used and analyzed during the current study is not expected to be shared. If someone wants to obtain the raw data of this study, a reasonable request can be sent to the first author by E-mail.

## Abbreviations

EFA: Exploratory factor analysis
CFA: Confirmatory factor analysis
PCA: Principal component analysis
KMO: Kaiser–Meyer–Olkin
SEM: Structural equation modeling
CFI: Comparative fit index
RMSEA: Root mean square error of approximation
AIC: Akaike Information Criterion
AGFI: Adjusted goodness-of-fit statistic
GFI: Goodness-of-fit index
TLI: Tucker–Lewis coefficient
CN: Critical N
