# Supplementary material for: Analysis of factors affecting students going to school toilets in a rural primary school in China
Source: BMC Public Health. 2021 Jan 6;21:32. doi: 10.1186/s12889-020-10099-4 (PMC7789347; doi:10.1186/s12889-020-10099-4)
Supplement: Supplementary file 1 — Additional file 1. Questionnaire. [file 12889_2020_10099_MOESM1_ESM.docx]

**Attachment 1:**

**Questionnaire on the Students’ Behavior of Using Toilet in Rural Primary School**

Gender: Age: Grade:

**Frequency of voiding and defecation**

1.How often did you defecate in school toilets?

a. Always b. Sometimes c. Never

2.How many times did you void in school toilets one day?

a.5-6 times per day b.3-4 times per day c.1-2 times per day d.≤1 times per day e. Never

**Toilet facilities**

1.1 Was the toilet far away?

a. Not far away b. A little far c. Far d. Very far e. Not notice

1.2 Was the time enough to use the toilet at break?

a. Enough b. A little enough c. Not enough d. Inadequate e. Not notice

1.3 Was the toilet usually crowded?

a. Never crowded b. Crowded sometimes c. Crowded always

d. Crowded every time e. Not notice

1.4 Did you need to wait while using the toilet?

a. Never need to wait b. Need to wait sometimes c. Need to wait always

d. Need to wait every time e. Not notice

1.5 Were you late for class due to using toilet at break?

a. Never b. Sometimes c. Always d. Every time e. Not notice

1.6 Were you criticized by a teacher due to using the toilet (such as, late for class for using toilet, wanted to using toilet during class)?

a. Never b. Sometimes c. Always d. Every time e. Not notice

**Toilet hygiene**

2.1 Was the toilet usually clean?

a. Very clean b. clean c. A little dirty, bearable

d. Very dirty, unbearable e. Not notice

2.2 Was there usually any stool or urine on the toilet floor?

a. Never existed b. Existed sometimes c. Existed always

d. Existed every time e. Not notice

2.3 Was there usually any garbage (such as toilet paper) on the toilet floor?

a. Never existed b. Existed sometimes c. Existed always

d. Existed every time e. Not notice

2.4 Was there usually any dirty water stain on the toilet floor?

a. Never existed b. Existed sometimes c. Existed always

d. Existed every time e. Not notice

2.5 Was there usually any stool or urine in the defecation pit?

a. Never existed b. Existed sometimes c. Existed always

d. Existed every time e. Not notice

2.6 Was there usually any garbage (such as toilet paper) in the defecation pit?

a. Never existed b. Existed sometimes c. Existed always

d. Existed every time e. Not notice

2.7 Was the toilet well ventilated and smell free?

a. Smelled good b. No smell c. A bit stinky, bearable

d. Very stinky, unbearable e. Not notice

2.8 Was the toilet usually dark?

a. Very bright b. light c. A bit dark

d. Dark e. Not notice

2.9 Did you have the experience of slipping or falling in the toilet?

a. Never b. Sometimes c. Always d. Every time e. Not remember

2.10 Had you ever accidentally stepped into the defecation pit in the toilet?

a. Never b. Sometimes c. Always d. Every time e. Not remember

2.11 Were there usually flies and maggots in the toilet?

a. No one b. A small amount c. A little more d. A lot of e. Not notice

2.12 Had you ever been bullied by other students in the toilet?

a. Never b. Sometimes c. Always d. Every time e. Not remember

**Hygiene practice**

3.1 Would you urinate or defecate on the toilet floor if in case of anurgency ?

a. Never b. Sometimes c. Always d. Every time e. Not remember

3.2 Would you endure waiting for the break if you wanted to use toilet during class?

a. Never b. Sometimes c. Always d. Every time e. Not remember

3.3 Would you litter your used toilet paper?

a. Never b. Sometimes c. Always d. Every time e. Not remember

3.4 Would you pay attention to the urine or feces at the designated location in the toilet?

a. Every time b. Always c. Sometimes d. Never e. Not notice

3.5 Did the teacher teach you how to wash your hands after using toilet?

a. Every time b. Always c. Sometimes d. Never e. Not notice

3.6 Do you wash hands every time after using the toilet?

a. Every time b. Always c. Sometimes d. Never e. Not notice

**Peer relationship**

4.1 How many close friends did you usually have?

a.≥5 friends b.3-4 friends c.1-2 friends

d. only one friends e. No one

4.2 Did you often go to the toilet alone?

a. Never b. Sometimes c. Always d. Every time e. Not notice

4.3 Did you usually go to the toilet with close friends?

a. Every time b. Always c. Sometimes d. Never e. Not notice

4.4 Would you accompany your close friends to the toilet whenever you did not want to go?

a. Every time b. Always c. Sometimes d. Never e. Not notice

4.5 Would you wait for your close friends to return to the classroom whenever you met him or her in the toilet?

a. Every time b. Always c. Sometimes d. Never e. Not notice

**Experience**

5.1 How did you usually deal with the situation of meeting a classmate having a bad relationship with you when going to the toilet?

a. Did not matter, still go to the toilet b. Did not go to the toilet sometime

c. Did not go to the toilet most of the time d. Did not go to the toilet every time

e. Not notice

5.2 How did you usually deal with the situation of meeting a classmate who liked to bully other students when going to the toilet?

a. Did not matter, still go to the toilet b. Did not go to the toilet sometime

c. Did not go to the toilet most of the time d. Did not go to the toilet every time

e. Not notice

5.3 How did you usually deal with the situation of meeting a classmate who liked to make fun on your going to the toilet?

a. Did not matter, still go to the toilet b. Did not go to the toilet sometime

c. Did not go to the toilet most of the time d. Did not go to the toilet every time

e. Not notice

5.4 How did you usually deal with the situation of meeting a teacher when going to the toilet?

a. Did not matter, still go to the toilet b. Did not go to the toilet sometime

c. Did not go to the toilet most of the time d. Did not go to the toilet every time

e. Not notice
